# Outcomes of Intramedullary Nailing Versus Plate Fixation of Humerus Shaft Fractures: A Single‐Center Retrospective Study

**DOI:** 10.1155/aort/8821939

**Published:** 2025-11-06

**Authors:** Asma Al Rasbi, Ayman Al Amri, Ahmed Al Hadeethi, Issam Al Aghbari, Osama Al Senani

**Affiliations:** ^1^ Orthopedics Division, Sultan Qaboos University Hospital, Seeb, Oman, squh.edu.om; ^2^ Ministry of Health, Muscat, Oman, moh.gov.om; ^3^ College of Medicine, Sultan Qaboos University, Seeb, Oman, squ.edu.om

## Abstract

The optimal fixation method for humeral shaft fractures remains a topic of debate. Intramedullary nailing (IM nailing) and plate fixation are the most common surgical options, each with distinct advantages and complications. This study aimed to compare the functional outcomes and complications associated with IM nailing and plating fixation for humeral shaft fractures at Sultan Qaboos University Hospital. This retrospective cohort study included adults who underwent humeral shaft fracture fixation from January 2012 to December 2022. Patients were treated with either IM nailing or plate fixation. Outcomes that were measured included time to union, operative time, complications rate, and the abbreviated form of the Disabilities of the Arm, Shoulder, and Hand (DASH) score. About 73 patients were included in the study, 37 of whom underwent IM nailing, and 36 patients had plate fixation. There were no statistically significant differences in any of the measured outcomes: surgical site infection (*p* = 0.475), operative time (*p* = 0.365), time to union (*p* = 0.055), delayed union (*p* = 0.325), nonunion (*p* = 0.491), revision procedures (*p* = 0.254), and postoperative radial nerve injury (*p* = 1.000). The Quick DASH scores were similar between the two groups (*p* = 0.443). In conclusion, both IM nailing and plating fixation are effective methods for treatment of humerus shaft fractures with no statistically significant differences. The Nail group showed not only a trend of slightly shorter time to union but also a higher rate of complications. The Plate group had a higher incidence of delayed union but fewer cases of postoperative complications. Surgical choice should be individualized based on patient and fracture characteristics.

## 1. Introduction

Humerus shaft fractures account for about 5% of all fractures [1]. The best surgical treatment for humeral shaft fractures is still debatable. However, the most common operative treatment options are intramedullary nailing (IM nailing) and plate fixation, each with its advantages and disadvantages. IM nailing is less invasive and may require less surgical time but may be associated with rotator cuff symptoms. Plating fixation enables precise anatomical reduction and fracture compression, but it carries a higher risk of radial nerve injury. Three recent meta‐analyses reported pooled rates for surgical infections, ranging from 1.6% to 2.3% after nailing and 1.7%–7.7% after plating. The rates of secondary nerve palsy were 2.5% to 6.4% after nailing compared to 2.9%–6.9% after plating. Nonunion rates ranged from 3.6% to 9.2% after nailing and 1.1%–8.6% after plating [2–4]. Functional outcome differences are also an important factor to consider, as it is always a point of debate whether avoiding rotator cuff complications with plating improves the functional outcomes. A recent prospective study looked at the DASH score in patients who underwent IM nailing for humerus shaft fracture and showed an average score of 16.51 reflecting a satisfactory function [5]. The HUMMER study compared the DASH scores in patients who underwent plating compared to nailing and showed no differences in the scores of both groups [6].

Sultan Qaboos University Hospital (SQUH) is a trauma center in Oman that favors the use of IM nailing for humerus shaft fractures. However, no previous studies have been carried out on the outcomes of this procedure in our population, which might differ from the international studies in view of different ethnic and surgical expertise factors. This study aimed to examine the differences between IM nailing and plating fixation on functional recovery and complications in adults with a humeral shaft fracture treated in SQUH.

## 2. Materials and Methods

### 2.1. Setting and Participants

This is a retrospective cohort study conducted at SQUH, which is a Level I trauma center located in the capital region in Muscat, Sultanate of Oman. We included cases presented to our hospital from January 1, 2012, to December 31, 2022, with a diagnosis of humerus shaft fractures after obtaining approval from the Institutional Medical Research ethical approval of SQU (reference: MREC #3536). All patients who were ≥ 18 years of age had a humeral shaft fracture confirmed by radiography and underwent a surgical procedure < 14 days after hospital presentation was included after they provided written informed consent. Patients with a pretrauma disability or additional trauma to the arm that could affect the outcome or with expected problems with maintaining follow‐up were excluded.

### 2.2. Surgical Intervention

Treatment was provided based on local protocols, and the surgical procedure was performed or supervised by certified, experienced orthopedic trauma surgeons at our institute. The decision about which implant to use was left to the discretion of the treating surgeon. IM nailing cases were done using the Expert Humeral Nailing System developed by DePuy Synthes, while plating procedures were done using narrow 4.5‐mm limited contact dynamic compression plates either with an anterolateral approach or posterior approach. The immediate postoperative protocol was similar in both groups and consisted of a fully active and passive range of motion (ROM) of the shoulder and elbow with restricted weightlifting for 3 weeks, followed by gradual resumption of daily living activity until reaching the baseline functional status.

### 2.3. Assessment and Follow‐Up

For all patients, the follow‐up visits were scheduled at 2 weeks (7–21 days), 6 weeks (4–8 weeks), 3 months (11–15 weeks), 6 months (5–8 months), and 12 months (12–14 months) postsurgery. Clinical data were collected from the patients’ medical files at each visit. Also, shoulder and elbow ranges of motion were measured clinically, and patients were asked about the level of pain and activity resumption during subsequent visits. Anteroposterior and lateral radiographs of the humerus were made at the presentation, after the operation, and at each follow‐up visit.

### 2.4. Statistical Analysis

Demographic data such as age, gender, and side, and time from injury to surgery and follow‐up time were collected from the hospital information system (HIS). The abbreviated form of the Disabilities of the Arm, Shoulder, and Hand (Quick‐DASH) score served as the primary outcome measure, which we recorded prospectively through clinic visits or phone calls.

The secondary outcome measures were the pain level (on a visual analog scale [VAS]), shoulder and elbow ROM, resumption of daily living activities, complications and associated secondary interventions, and radiographic healing. Nonunion was defined as a failure to heal at 6 months postoperatively with no progress toward healing seen on radiographs [7]. Data were analyzed using the Statistical Pfor the Social Sciences (SPSS) Version 26 (IBM). All statistical tests were 2‐sided, and analysis was by intention to treat. Missing data were not imputed. Categorical data were analyzed using the chi‐square test. Continuous data, which were all non‐normally distributed according to the Shapiro–Wilk test, were analyzed using the Mann–Whitney *U* test. Significance was set at *p* < 0.05.

## 3. Results

### 3.1. Patients and Treatment Details

This study included 73 cases, 37 (50.7%) patients in the Nail group and 36 (49.3%) patients in the Plate group. The results are shown in Table 1. The mean age of patients in the Nail group was 37.8 ± 11.8 years, while in the Plate group, it was 31.2 ± 13.4 years. Gender distribution was similar between the groups, with males being predominant in both (70.3% in the Nail group and 72.2% in the Plate group, *p* = 0.530). According to the AO classification system, a higher percentage of Type A fractures in the Nail group (56.8%) compared to the Plate group (41.7%). Type B fractures were more common in the Plate group (41.7%) compared to the Nail group (24.3%). Type C fractures were rare in both groups, with a slightly higher occurrence in the Plate group (13.9% vs. 2.7%, *p* = 0.084). The mean days to surgery were similar between the groups (5.3 ± 8.7 days for the Nail group and 5.3 ± 5.3 days for the Plate group, *p* = 0.252). The duration of surgery was also comparable (140 ± 35.7 min for the Nail group and 146.0 ± 54.5 min for the Plate group, *p* = 0.365).

**Table 1:** Demographics, time to union, and complication rate in both groups.

Variable	Nail group % (*n*) Total = 37	Plate group % (*n*) Total = 36	*p* value
Age (mean)	37.8 ± 11.8	31.2 ± 13.4	0.030^a^
Gender			0.530^b^
Female	29.7% (11)	27.8% (10)	
Male	70.3% (26)	72.2% (26)	
Side			0.352^b^
Left	54.1% (20)	41.7% (15)	
Right	45.9% (17)	58.3% (21)	
AO class			0.084^b^
A	56.8% (21)	41.7% (15)	
B	24.3% (9)	41.7% (15)	
C	2.7% (1)	13.9% (5)	
Days to surgery (mean in days)	5.3 ± 8.7	5.3 ± 5.3	0.252^c^
Duration of surgery (mean in minutes)	140 ± 35.7	146.0 ± 54.5	0.365^c^
Time to union (mean in weeks)	10.69 ± 8.2	12.1 ± 6.5	0.055^c^
Follow‐up time (mean in months)	14.7 ± 15.3	10.0 ± 10.2	0.228^c^
Complications	31.0% (9)	13.3% (4)	0.125^d^
SSI	3.6% (1)	0	0.475^d^
Delayed union	3.6% (1)	11.1% (3)	0.352^d^
Nonunion	7.1% (2)	0	0.491^d^
Post op radial nerve palsy	2.7% (1)^e^	0	1.000^d^
Revision or implant removal	17.2% (5)	6.7% (2)	0.254^d^
Shoulder pain or reduced ROM	17.2% (5)	10.0% (3)	0.472^d^
Quick DASH score (mean)	20.3 ± 30.1	18.4 ± 18.2	0.443^c^

^a^Independent sample *t*‐test.

^b^Chi‐square test.

^c^Using Mann–Whitney *U* test, as measure of normality showed non‐normal distribution of the variables.

^d^Fisher’s exact test.

^e^Radial nerve palsy recovered.

### 3.2. Functional Outcome and Complications

The mean time to union was slightly shorter in the Nail group (10.69 ± 8.2 weeks) compared to the Plate group (12.1 ± 6.5 weeks, *p* = 0.055). The follow‐up time was longer in the Nail group (14.7 ± 15.3 months) compared to the Plate group (10.0 ± 10.2 months, *p* = 0.228) but not statistically significant.

We were able to measure the Quick DASH score in 15 patients in the Nail group, and 12 patients in the Plate group, with similar scores between the groups (20.3 ± 30.1 for the Nail group and 18.4 ± 18.2 for the Plate group, *p* = 0.443). This outcome measure is limited by the low response rate of the patients (40.5% response rate in the Nail group, and 33.3% in the Plate group).

**Figure 1:**
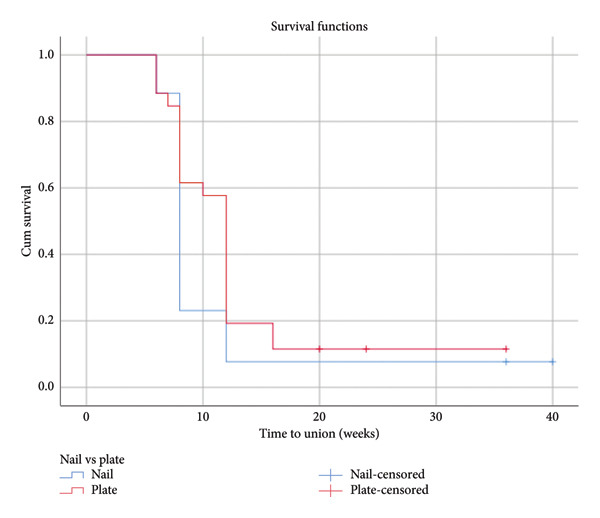
Kaplan–Meier curve for time to union.

Complications were more frequent in the Nail group (31.0%), with 3.6% experiencing surgical site infections (SSI), 3.6% delayed union, 7.1% nonunion, 2.7% postoperative radial nerve palsy, 17.2% requiring revision or implant removal, and 17.2% experiencing shoulder pain or reduced ROM. In the Plate group, 11.1% had delayed union, 6.7% required revision or implant removal, and 10.0% shoulder pain or reduced ROM, with no cases of SSI, nonunion, or postoperative radial nerve palsy. Figure 1 is the Kaplan–Meier curve representing time to union. The differences in complications were not statistically significant. This study had patients who lost follow‐up. Some patients had postoperative documentation of radial nerve function but lost follow‐up after hospital discharge (9 patients in the Nail group and 6 patients in the Plate group), leaving no data on other primary or secondary outcomes. Additionally, a few patients had follow‐up visits at 2 and 6 weeks, with surgical site complications documented, but the follow‐up period was insufficient to assess union time or other complications (1 patient in the Nail group and 2 patients in the Plate group). These patients were excluded from the analysis of outcomes that could not be measured due to incomplete follow‐up.

## 4. Discussion

Despite the extensive international research done on the two methods of humerus shaft fracture fixation, controversy is still present. This was the first local study that aimed to look into our own local data and results compared with the existing literature. The study included 73 patients who sustained humerus shaft fractures and were treated surgically at SQUH, which is a Level I trauma center located in the center region in Muscat, Sultanate of Oman. This study compared the outcomes of humerus nail fixation using nails versus plates regarding patient demographics, surgical details, and postoperative outcomes. The results indicated that while both methods are effective, there are notable differences in certain aspects. The Nail group showed not only a slightly shorter time to union but also a higher rate of complications. The Plate group had a higher incidence of delayed union but fewer cases of postoperative complications such as radial nerve palsy and revision surgeries.

In our study, the mean time to union was slightly shorter in the Nail group (10.69 ± 8.2 weeks) compared to the Plate group (12.1 ± 6.5 weeks, *p* = 0.055), suggesting a potential advantage of nail fixation in terms of faster healing. Previous studies have also explored the efficacy of nail fixation for humerus fracture union. McCormack et al. found that nail fixation resulted in shorter operative times and faster union rates than plate fixation [8]. Furthermore, Mocini et al. found that newer generation straight humeral nails allow faster bone healing and better functional outcomes at mid‐term follow‐up [9].

However, the Nail group experienced a higher rate of complications, including surgical site infections (SSI), delayed union, nonunion, postoperative radial nerve palsy, and shoulder pain or reduced ROM. Although these differences were not statistically significant, they highlight the need for careful consideration of potential risks when choosing the fixation method. In our study, the Nail group had higher revision or implant‐related complications with a rate of 17.2% (7 cases), while the Plate group had only two cases (6.7%) of revision or implant removal. The risk of a technical error appears higher after nailing. The main indication for revision surgery in this group was nail protrusion, which may explain the inferior shoulder function and ROM in this group. This finding aligns with the previous studies that have reported higher rates of implant‐related complications and subsequent removal in patients treated with intramedullary nails. Van Bergen et al. found that intraoperative complications and implant failures were more frequent in the nailing group compared to the plating group [10]. Also, a meta‐analysis by Beeres et al. 2022 shows a significantly higher revision rate after nailing (odds ratio [OR], 0.29; *p* = 0.02) [2].

Furthermore, in our study, the nailing group had a higher nonunion rate of 7.1% (2 cases), while the Plate group had no cases of nonunion. The nonunion rate of 9.0% after nailing was reported by van de Wall et al. and 10.1% by Den Hartog et al. which were in line with our study [3, 6]. However, two recent meta‐analyses showed no significant difference in nonunion rates between plating (3.0% and 5.6%) and nailing (4.3% and 6.9%) [2, 4]. Our study findings suggest that while nail fixation may offer faster healing, it comes with a higher risk of complications. Plate fixation is a safer option with comparable functional outcomes. Hence, the choice between nail and plate fixation should be individualized based on patient characteristics, fracture type, and potential risks. Surgeons should weigh the benefits of faster healing with nail fixation against the higher complication rates.

This study has several limitations, including a small sample size and missed follow‐up visits. Additionally, the retrospective nature of the study introduces potential biases, such as selection bias and recall bias, as well as bias in the surgeon’s choice of fixation method. The low response rate to the Quick DASH questionnaire further compromises the validity of the results. Future research should aim to address these limitations and explore the long‐term outcomes of both fixation methods.

## 5. Conclusion

Both nailing and plating fixation methods are effective for treating humerus shaft fractures, with nailing showing a slightly shorter time to union but also a higher rate of complications. While plating has a higher incidence of delayed union but fewer cases of postoperative complications such as radial nerve palsy and revision surgeries. The choice of method should be tailored to the individual patient’s condition, specific fracture characteristics, and surgeon’s own experience. Continued innovation in this field could improve outcomes and reduce complication rates for patients with humerus shaft fractures. Larger multicenter, prospective trials are needed to address the limitations in this study.

## Ethics Statement

Ethical approval was obtained on October 17th, 2024, MREC #3536.

## Conflicts of Interest

The authors declare no conflicts of interest.

## Author Contributions

Asma Al Rasbi: research design, the acquisition, analysis and interpretation of data, and paper drafting.

Ayman Al Amri: research design, supervision, and paper writing and review.

Ahmed Al Hadeethi: research design and data acquisition.

Issam Al Aghbari: data collection.

Osama Al Senani: data collection.

## Funding

The authors received no specific funding for this work.

## Data Availability

The data that support the findings of this study are available on request from the corresponding author. The data are not publicly available due to privacy or ethical restrictions.

## References

[1] Pidhorz L. , Acute and Chronic Humeral Shaft Fractures in Adults, Orthopedie Traumatologie: Surgery & Research. (2015) 101, no. 1, S41–S49, 10.1016/j.otsr.2014.07.034, 2-s2.0-84923105525. 25604002

[2] Beeres F. J. P. , van Veelen N. , Houwert R. M. et al., Open Plate Fixation Versus Nailing for Humeral Shaft Fractures: A Meta-Analysis and Systematic Review of Randomised Clinical Trials and Observational Studies, European Journal of Trauma and Emergency Surgery. (2022) 48, no. 4, 2667–2682, 10.1007/s00068-021-01728-7. 34219193

[3] van de Wall B. J. M. , Baumgärtner R. , Houwert R. M. et al., MIPO Versus Nailing for Humeral Shaft Fractures: A Meta-Analysis and Systematic Review of Randomised Clinical Trials and Observational Studies, European Journal of Trauma and Emergency Surgery. (2022) 48, no. 1, 47–59, 10.1007/s00068-020-01585-w. 33452548

[4] Wen H. , Zhu S. , Li C. , Chen Z. , Yang H. , and Xu Y. , Antegrade Intramedullary Nail Versus Plate Fixation in the Treatment of Humeral Shaft Fractures: an Updated meta-analysis, Medicine. (2019) 98, no. 46, 10.1097/md.0000000000017952. PMC6867742 31725653

[5] Naveen Kumar Reddy C. V. , Naik S. , and Biradar R. K. , Functional Outcomes of Intramedullary Interlocking Nailing for Humeral Shaft Fractures: A Prospective Study, Cureus. (2025) 17, no. 4, 10.7759/cureus.82550. PMC12088705 40390752

[6] Den Hartog D. , Mahabier K. C. , Van Bergen S. H. et al., Functional and Clinical Outcomes After Plate Osteosynthesis Versus Intramedullary Nailing of a Humeral Shaft Fracture: The Results of the HUMMER Multicenter, Prospective Cohort Study, Journal of Bone and Joint Surgery. (2023) 105, no. 14, 1101–1111, 10.2106/jbjs.22.00647. 37220192

[7] Anglen J. O. , Archdeacon M. T. , Cannada L. K. , and Herscovici D. , Avoiding Complications in the Treatment of Humeral Fractures, Journal of Bone and Joint Surgery American Volume. (2008) 90, no. 7, 1580–1589. 18594109

[8] McCormack R. G. , O’Brien P. J. , Buckley R. E. , McKee M. , Powell J. , and Schemitsch E. , Fixation of Fractures of the Shaft of the Humerus by Dynamic Compression Plate or Intramedullary Nail: A Prospective, Randomized Trial, The Journal of Bone & Joint Surgery. (2000) 82, no. 3, 336–339. 10813165 10.1302/0301-620x.82b3.9675

[9] Mocini F. , Rovere G. , De Mauro D. , De Sanctis E. G. , Smakaj A. , Maccauro G. , Liuzza F. , and Liuzza F. , Newer Generation Straight Humeral Nails Allow Faster Bone Healing and Better Functional Outcome at Mid-term, Journal of Orthopaedic Surgery and Research. (2021) 16, no. 1, 10.1186/s13018-021-02776-w. PMC8529842 34670577

[10] Van Bergen S. H. , Mahabier K. C. , Van Lieshout E. M. M. et al., Humeral Shaft Fracture: Systematic Review of Non-Operative and Operative Treatment, Archives of Orthopaedic and Trauma Surgery. (2023) 143, no. 8, 5035–5054, 10.1007/s00402-023-04836-8. 37093269 PMC10374687

